# Supporting someone with cancer during the COVID‐19 pandemic: A mixed methods analysis of cancer carer's health, Quality of Life and need for support

**DOI:** 10.1111/hsc.13768

**Published:** 2022-03-04

**Authors:** Olinda Santin, Julie Mc Mullan, Chris Jenkins, Lesley A. Anderson, Charlene M. Mc Shane

**Affiliations:** ^1^ School of Nursing and Midwifery Queen's University Belfast Belfast UK; ^2^ Centre of Public Health Queens University Belfast Belfast UK; ^3^ School of Medicine Medical Science and Nutrition University of Aberdeen Aberdeen Scotland

**Keywords:** cancer, caregivers, carers, COVID‐19, health status, mental health, oncology, Quality of Life

## Abstract

The COVID‐19 pandemic has greatly affected the delivery of cancer care. Due to social restrictions and reductions in health service contact, it is expected that the burdens experienced by informal carers have risen. This study provides an analysis of cancer carer's experiences and needs as a consequence of the pandemic. An online mixed method design was used. The survey included open‐ended responses to explore carer's experiences and measures of health status (EQ‐5D‐5L), Quality of Life (WHOQoL‐BREF) and impact of COVID‐19. Open‐ended responses were analysed thematically according to Miles and Huberman techniques and quantitative data were analysed descriptively. One hundred and ninety‐six cancer carers participated in the online survey. Mixed method analysis demonstrated that carers were experiencing major difficulties. Of these *n* = 142/72.4% experienced challenges related to anxiety and depression; 35.2% rated these problems as slight with 25% rating these as moderate and 11.2% as severe. Qualitative analysis identified significant and sustained negative impacts of the pandemic on psychological health, social isolation, finance and access to health services with carers requiring urgent information and support. Carer's challenges have deepened throughout the COVID‐19 pandemic. There is an urgent need to develop innovative ways to provide support for carers to provide palliative and supportive care at home now and during recovery from the pandemic. Due to the need for infection control meaningful development and integration of urgent digital technology might be the most feasible solution.

**What is known about this topic?**

- COVID‐19 and the associated restrictions have had a significant impact on the delivery of cancer care.
- Many patients with cancer have been shielding and experiencing reduced face‐to‐face contact with healthcare professionals.
- The pressure of a backlog and potential ongoing social distancing increases the need to rapidly develop an understanding of the impact of COVID‐19 on patients with cancer and their families.

**What this paper adds:**

- The COVID‐19 pandemic has had a negative impact on cancer carers, particularly regarding their mental health.
- Burden of care has become great and sustained and is related to the ongoing impact of COVID‐19 on disrupting cancer services; provision of care in isolation; lack of other social support and heightened information needs.
- There is an urgent need for the development of online support for cancer carers to mitigate some of the wider challenges such as social isolation and loneliness identified by many carers.

## 1 INTRODUCTION

The World Health Organization (WHO) declared a global pandemic of novel coronavirus (COVID‐19) infection on 11 March 2020 (WHO, 2020). In response to the pandemic, countries around the globe introduced a range of measures involving restriction of movement, social distancing and closures of amenities to suppress the spread of the virus (Han et al., 2020). Healthcare systems experienced dramatic changes to normal service with staff, beds and clinics reassigned to assist in the management of COVID‐19 (Lewis & Roques, 2020). These changes and restrictions are likely to have a significant impact on patients with cancer and carers. In many countries, cancer services have been forced to make difficult decisions regarding COVID‐19 exposure risk and the risk of cancer treatment delay (Ueda et al., 2020). Multiple hospital visits and immunosuppression meant that patients with cancer were viewed to be particularly vulnerable to COVID‐19 (Ueda et al., 2020). Healthcare services rapidly changed to remote appointments with changes to surgery and chemotherapy provision (Lewis & Roques, 2020).

With many patients with cancer shielding and experiencing reduced face‐to‐face contact with healthcare professionals, it is expected that strain on informal carers has risen considerably (Egan, 2020). As recovery from the pandemic continues and carers around the globe experience varying degrees of restrictions, it is paramount that healthcare services gain an understanding of the needs and issues faced by carers to ensure that services are appropriately adapted and provided.

Prior to the outbreak, the burden of cancer on informal carers was already demonstrated to be significant (Santin et al., 2014; Stenberg et al.^,^ 2010). In the context of COVID‐19, many families have been forced to further manage severe complications and healthcare needs, with minimal health knowledge or skills and with limited healthcare professional input (Chan et al., 2020). Although some hospitals and treatments have had periods of re‐commencing some services, the pressure of a backlog, ongoing lockdowns and vaccination roll out increase the need to rapidly develop an understanding of the impact of COVID‐19 on carers and how they can be supported remotely. This study aims to provide an overview of cancer carer's health and experiences during the COVID‐19 pandemic and present their perspectives on the support they require.

## 2 METHODS

### 2.1 Design

A mixed method approach using an online survey was chosen to understand the impact of COVID‐19 on cancer carers during the pandemic between April and August 2020. Ethical approval was provided by Queen's University Belfast Ethical Committee. The online survey was developed by the expert team and in consultation with the literature. The survey was reviewed by several healthcare professionals and expert patients with several changes made in relation to readability.

### 2.2 Data collection

The survey was hosted by SurveyMonkey (www.surveymonkey.co.uk) and included closed‐ and open‐ended questions. Respondents were asked to complete demographic information (15 questions), the EQ‐5D‐5L (EUROQOL) for quality‐of‐life findings and the WHO‐QoL‐BREF (The WHOQOL Group, 1998). Thirteen closed questions developed by the team explored carer care‐giving duties, changes experienced during the pandemic, challenges faced and what support would be useful moving forward. Three open‐ended questions regarding overall all experiences, difficulties and challenges and needs for support were also included. Carers were defined as anyone over 18 years old who provide unpaid (except state benefits) informal care for any person currently diagnosed with cancer, at any stage of disease. The survey was promoted via multiple social media platforms online including a study‐specific Twitter and Facebook account, local media outlets and through charitable cancer and carer organisations globally.

### 2.3 Analysis

The data were extracted from the SurveyMonkey platform and analysis undertaken using Stata MP v15.1 for quantitative data and Microsoft Excel for qualitative assessment. Descriptive statistics were used to describe the data. The WHO‐QoL BREF (The WHOQOL Group, 1998) and EQ‐5D‐5L (EURO‐QOL) were analysed according to published methods with findings presented as proportions and median as appropriate. Due to numbers, the EQ‐5D‐5L dimensions were dichotomised to 'no problems' versus 'any problems'.

Open‐ended responses were managed via NVIVO software and analysed using thematic analysis according to Miles and Huberman techniques (Miles & Huberman, 1994). Themes were inductively identified after familiarisation with data and were agreed upon by the research team. The content was manually coded into identified themes and cross‐checked and validated by two researchers. The researchers counted frequencies in the open‐ended question data, as well as identifying quotes that were representative of themes emerging throughout the study. Data were further triangulated against quantitative data to ensure validity.

## 3 FINDINGS

A total of 196 cancer carers completed survey and open‐ended responses of which the majority were female (*n* = 164, 83.7%), resided in the UK (*n* = 168, 86%) and had been a carer for 2–5 years (*n* = 68.2, 37.5%) and were employed (*n* = 120, 61.2%), (Table 1).

**TABLE 1:** Demographics of survey respondents

	Number (%) (*N* = 196)
Gender	
Male	32 (16.3)
Female	164 (83.7)
Age (years)	
≤50	99 (50.5)
>50	97 (49.5)
Country of Residence	
UK	168 (85.7)
Other	28 (14.3)
Residence	
Rural area	81 (41.3)
Urban area	115 (58.7)
Marital status	
Married/cohabiting	159 (81.1)
Other^a^	37 (18.9)
Children aged 18 years or younger living at home	
Yes	55 (28.1)
No	141 (71.9)
Highest level of education	
University qualification	117 (59.7)
Other	79 (40.3)
Employment status	
Employed	120 (61.2)
Unemployed/Retired/Carer	76 (38.8)
In receipt of financial support for caregiver role	
No financial support	164 (85.4)
Government support/paid for care‐giving services	28 (14.6)
*Missing*	4
Length of time as a caregiver	
Less than 2 years	59 (30.7)
2–5 years	72 (37.5)
>5 years	61 (31.8)
*Missing*	4
Comorbidity (self‐reported)	
Yes	77 (39.3)
Not reported	119 (60.7)

^a^ Single/separated/Divorced/Widowed

At survey completion, cancer carers rated their health as 80 out of 100 on the EQ‐5D visual analogue scale (median, interquartile range: 62.5–90). Most carers reported no problems with mobility (*n* = 155, 79.1%) or self‐care (*n* = 178/90.8%). One‐third of carers (*n* = 66/33.7%) experienced some problems with usual activities with *n* = 35/17.9% rating these problems as slight and *n* = 24/12.2% as moderate. A higher proportion of difficulties were reported in domains for pain and discomfort (54.1%/*n* = 106) with 36.7% (*n* = 72) reporting these problems to be slight, 11.2% (*n* = 22) moderate and 5.6% (*n* = 11) severe. Just under three quarters of carers (*n* = 142/72.5%) experienced problems in relation to anxiety/depression, with 35.2% (*n* = 69) rating these problems as slight, 25% (*n* = 49) moderate and 11.2% (*n* = 22) severe. Within the WHO‐QOL‐BREF questionnaire, 10.2% (*n* = 20) of cancer carers reported their general quality of life as being poor/very poor, while 16.3% (*n* = 32) reported being dissatisfied/very dissatisfied with their general health.

Thematic analysis of open‐ended responses identified five key themes which included: the psychological impact of providing informal care during COVID‐19, the social impact on available social support and the impact on employment, the increase in caregiver burden including the increase of tasks, limited support and difficulties with obtaining essential items, living with the impact on cancer and palliative services and support requirements. The qualitative analysis supported the quantitative findings that COVID‐19 was having a negative impact on cancer carers.

### 3.1 Psychological experience

A key theme identified by carers was the negative psychological impact of caring for someone with cancer during the COVID‐19 pandemic. This supports the quantitative findings discussed. Carers reported experiencing intense fear that the person with cancer would contract COVID‐19 and die. Continuous worry about the person with cancers survival was described as having a negative impact on levels of anxiety and mood.

'I'm constantly frightened and anxious, I constantly worry about what will happen'

'I'm living in isolation with my fear that my husband will get this (COVID‐19) and die'

'The fear should he get the virus and his ability to survive it.'

Carers reported living in a state of hypervigilance regarding the potential transmission of COVID‐19 and felt frustrated and frightened regarding the relaxed preventative behaviours of others. This is supported with 96.7% of carers reporting concern about passing on COVID‐19 and 43.4% reporting extreme concern about it. Carers discussed living in the fear that the patient would be admitted to the hospital and potentially die alone without the company of loved ones. In addition to living with fear and anxiety, carers reported that social distancing had resulted in a lack of contact with family and friends, which, in turn, reduced the potential of any respite or support with cancer caring responsibilities. Carers discussed that phone calls and video calls had helped them to feel supported, but they were not equal to face‐to‐face contact.

I care for my daughter who has cancer. I'm worried about her a lot, really worried. I'm worried also because she had only video call check up with her oncologist. So no bloods taken. I'm extremely worried that if she gets coronavirus, disease could be severe one for her with complications and she could need hospital or die'.

### 3.2 Social impact

Open‐ended responses identified additional social issues not measured by the quantitative survey. A key theme identified was the negative social impact of social distancing restrictions on carers. Carers reported that social distancing restrictions greatly reduced their support network, which they relied upon to help them cope with and manage their caring role. Cancer carers reported feelings of loneliness and isolation with many having only the person they were caring for as company.

'Very lonely. I miss normal human contact'.

The closure of recreational activities such as gyms, swimming pools and cafes resulted in carers being unable to rely on their usual coping mechanisms and as a result, negatively affected mental health. In addition, restrictions on society were viewed as 'stealing time' from families with many feeling that the precious time that the person they were caring for had left was wasted.

Can't see our sons face to face, had to cancel holidays, meetings friends, hobby meetings with friends, confined to the house for a lot of the time, courses for carers have been cancelled, exercise classes cancelled.

'We are both social people with a lot of hobbies and friends which we both miss very much. Especially difficult for my husband who has a terminal illness and is feeling well at the moment, and we feel we could be making the most of this window during his illness'.

'It is mentally difficult to be a home.'

'Because of cancer, we don't have much time left, our time is precious, we wanted to do things and we can't'

Carers reported a significant negative impact of COVID‐19 on employment, working patterns and finances. In support of this, 42.9% of carers reported that they had experienced financial burden due to COVID‐19. Some carers reported a leave of absence from work to shield the person they were caring for and as a result, they were experiencing significant financial worry and stress. This was a particular concern for those self‐employed carers. In addition to the financial pressures, working unsociable hours to keep up with the demands of their job and caring responsibilities.

'Working from home and doubling of my workload means I'm often working longer days and at weekends to catch up.'

Being furloughed has had an impact on me for financial and well‐being reasons. The most difficult thing has been trying to provide care safely for my mum who has stage 4 lung cancer and is about to undergo therapy ‐ it has been very scarey and upsetting at times.

### 3.3 Caregiver burden

The thematic analysis demonstrated that carers reported a greater sense of burden and increased caring responsibilities as a consequence of COVID‐19. Increased burden was described in association with the reduction of respite and supportive services, complexity in obtaining essential items such as groceries and medications, working from home and homeschooling children. This is supported in quantitative findings demonstrating that 52.2% of carers experienced difficulties receiving delivery slots. This additional burden was described as leading to extreme exhaustion for many carers.

Trying to secure shopping delivery slots has been stressful with no assistance from outside organisations. Couldn't get deliveries to begin but did manage to click & collect. Husband had Plenty of letters, text messages, phone calls but no clear help with how to get shopping delivered.

Carers with children and those in employment reported additional burden as they attempted to manage multiple tasks associated with caring for children at home, managing employment and homeschooling. Many carers described feeling overwhelmed and unable to cope.

'The day care and respite care were valuable to both of us, without it I have the full burden and cannot cope'.

'Working, keeping house, childcare, advocating for my mother ‐ juggling it all…'.

I'm reducing my working hours because of all the caring responsibilities

### 3.4 Impact on cancer and palliative services

Survey findings demonstrated that 59.6% (*n* = 109) carers reported that cancer treatment for the person they cared for had not been affected, 24.6% (*N* = 45) reported treatment delays and 15.8% (*N* = 29) that treatment was unavailable. Open‐ended responses provided further insight and highlighted service disruption in relation to delayed cancer appointments, tests and treatments. As a result of these disruptions, carers reported experiencing intense worry regarding the long‐term impact of delays on the health and survival of the person they cared for.

'My mother will not be able to receive the palliative chemotherapy treatment to prolong her life and improve her system and quality of life. If she catches covid she will die sooner'

'I worry that she will get COVID before she can start her treatment'

For those who continued treatment, carers reported that they could not attend appointments due to hospital restrictions and as consequence felt ill informed and worried that important information would be missed. Overall carers reported a lack of communication and information from cancer services to assist them and the person they cared for to manage the disease during the pandemic.

'For me, I worry that I don't have enough information regarding the cancer my husband has and how to deal with the emotions and symptoms confidently. COVID‐19 prevents me from going into the oncology office during appointments, so I am doing research via support groups'.

Carers supporting patients at the end of life reported reduced or withdrawal of supportive services and reported the difficulties surrounding managing the complexities of providing end‐of‐life care and the difficulties of doing this alone with no support.

'Better communication for patients following a cancer diagnosis, Better shared care as no one was reviewing my mother's medication for brain mets and her condition was deteriorating'

### 3.5 What carers want and need

About 61.4% of carers reported that they were unable to access COVID information regarding their caring role and that this would have been helpful. The thematic analysis provides further insights and suggests that carers had a number of needs for support. Carers discussed that they wanted tailored information and communication regarding shielding, accessing support, keeping virus free and caring for someone with cancer.

'We need clearer guidelines around social distancing and person who is terminally ill balance between quality of life, meeting the needs of patient with cancer as a result of shielding (e.g., shopping) and social distancing measures’.

Carers felt abandoned by support services and stated that they required online support including networking and counselling. Better communication from the government in terms of COVID‐19 and greater communication from cancer care professionals regarding cancer treatments. Carers suggested that clear, consistent information and guidance specific to cancer, in an accessible format, in one location would be extremely helpful. To improve social interaction for cancer carers, carers requested remote socialising via online support groups, counselling or even virtual activities such as exercise classes.

‘We need support, from other carers and professionals, online support groups or counselling would really help'.

Respondents also expressed their desire for more clarity around achieving a balance of caring for the individual while enabling them to have a reasonable quality of life. Carers particularly requested information regarding future hospital appointments and treatment, duration of shielding, provision of care should the caregiver become ill, keeping free from COVID‐19 and practicalities surrounding back‐to‐school arrangements. Emotional and financial support were also areas highlighted as requiring urgent support.

More face to face interaction with healthcare professionals even if that is through video conferencing (Zoom, Skype, etc.)

The commencement of respite services and carer support within the home was viewed as a priority to provide carers with support. Seventy percent of carers reported that they did not know, or were unsure, how to access carer support. It was suggested that the Government should consider providing respite care for carers or allow another family member to support them in their demanding role.

### 3.6 Discussion

This study demonstrates that the COVID‐19 pandemic has had a negative impact on cancer carers, particularly regarding their mental health. A systematic review highlighted the impact of the COVID‐19 pandemic on the general population reporting high rates of symptoms of anxiety (6.33% to 50.9%), depression (14.6% to 48.3%) and psychological distress (34.43% to 38%) (Xiong et al., 2020). Our study indicates that the impact of the pandemic on carers may be even more significant. Carers reported that their burden of care had increased significantly from the beginning of the pandemic with 72.5% of the carers reporting having experienced psychological problems with over half of these describing these problems as moderate to severe. While studies on the needs of cancer carers consistently point to the significant impact that caring has on mental and psychological health (Girgis et al., 2013; Oberoi et al., 2016). Findings would indicate that many carer's challenges have deepened throughout the COVID‐19 pandemic, particularly those with young children.

Reasons for the increased burden of care may relate to the impact of COVID‐19 on disrupting cancer services; provision of care in isolation; lack of other social support and heightened information needs. Our findings demonstrated that over 60% of carers require specific support and information regarding how the pandemic has impacted on their care‐giving role. Given that carer's anxiety and depression are often associated with unmet information needs ^(^Oberoi et al., 2016), there is a need to address this information deficit.

Findings reported here further demonstrated that COVID‐19 and physical and social restrictions have negatively affected the perceived availability of support for carers. This reduction of support is likely associated with psychological problems identified as social distancing protocols have restricted usual support systems of carers. Social isolation, because of lockdowns, was reported in our study to lead to loneliness. In addition to the need for social support, findings demonstrate that financial strains are a major concern for cancer carers. Carers discussed the employment and financial consequences of self‐isolation due to the need to shield patients with cancer from COVID‐19.

Carers in financial difficulties need easy‐to‐access opportunities to discuss their challenges with social workers or known support networks for expert advice. Consideration should also be given to the mechanisms by which carers can access increased financial support as they recover from the pandemic.

It is common that cancer carers report poorer physical QOL compared to their peers (Santin et al., 2014) with many carers having comorbidities themselves (Backhaus et al., 2012; Torimoto‐Sasai et al., 2015). Our research showed that caring while living with comorbidities was the most consistent significant factor impacting on carers QOL. Specific research and support should be explored and developed for this group, given the heightened nature of their needs.

Carer's first point of contact for support is normally healthcare providers, particularly the general practitioner. Given restrictions on face‐to‐face consultation and prioritisation of patients with severe and urgent needs, alternative support mechanisms should be developed. Cancer carers identified the need for increased online counselling to help assist them to cope during the pandemic. Online support has been shown to benefit mental health in the general population (Tay et al., 2018) as well as for families affected by cancer (Santin et al., 2019). It is likely that online support would also benefit carers, although there is a lack of specific literature on this issue. During the COVID‐19 pandemic, screening for psychological issues could provide a quick referral process for cost‐effective psychological interventions via online platforms.

The development of online support more generally may be an area for exploration in mitigating some of the wider challenges highlighted in this study, such as social isolation and loneliness identified by many carers (Egan, 2020). Reliable and easy‐to‐access online information, which is also easy to update given the rapidly changing context of COVID‐19, may help alleviate information needs and associated stresses and anxieties experienced by carers. Given the importance of the relationship between carer health and patient outcomes, significant attention should be paid to supporting carer's mental health ^(^Bevans & Sternberg, 2012; Kim et al., 2008; Segrin & Badger, 2014).

Carers providing support to terminal patients with cancer reported the need for support and assistance as they manage the complexities of the patient's illness at home with many doing so in isolation.

There is a need to explore ways to provide palliative and supportive care at home in a safe way. In addition to reducing isolation and supporting mental health, digital and online solutions may be utilised to provide information, training and support during palliative care. Consideration should also be given to teleconsultation for personalised training of carers or regular telephone calls which may assist carers to feel less isolated and better supported.

Study findings should be interpreted with caution given the relatively small caregiver sample and the majority of carers participating from the UK. The nature of the sample presents limitations in the generalisability of the findings; this is particularly apparent for the high proportion of carers with a university degree. Due to the online sampling method used, it is also likely that this survey is only representative of those carers who are comfortable accessing and using resources online supporting the need to assess the needs of careers who do not access the Internet or from a range of educational levels.

Urgent engagement with health and supportive care providers particularly primary care, cancer services and the voluntary sector is required to identify feasible solutions in currently strained services. This should be considered with the understanding that many face‐face consultations are expected to last no more than 15 min to reduce risk of transmission; therefore, professionals are likely to be constrained by time to support a carer with complex issues. This further indicates the need to consider developing online and digital supports for carers and the role that the voluntary sector could play to support the ongoing need. It should also be noted that online support may not benefit all carers, particularly those less comfortable using technology. For these carers, other remote solutions, such as greater support over the phone, should be considered. While phone support is harder to scale and more resource intensive, this may still be required for some carers.

The COVID‐19 pandemic has added additional burdens and challenges for many people caring for someone with a cancer diagnosis. Digital technologies may offer solutions to meet the information, training and support needs of carers, while reducing the risk of COVID‐19 transmission.

## CONFLICTS OF INTEREST

None.

## AUTHOR CONTRIBUTIONS

OS, JMc M, L Anderson and C Mc Shane were involved in data collection. All authors contributed to the analysis of data and the drafting of the manuscript.

## Data Availability

The data generated during this study are available from the corresponding author on reasonable request.
